# The Athletic Intelligence Quotient and Performance Outcomes in Professional Baseball

**DOI:** 10.3389/fpsyg.2021.629827

**Published:** 2021-06-24

**Authors:** James Kenneth Bowman, R. Thomas Boone, Scott Goldman, Alex Auerbach

**Affiliations:** ^1^Great Neck Public Schools, Great Neck, NY, United States; ^2^College of Arts and Sciences, University of Massachusetts - Dartmouth, North Dartmouth, MA, United States; ^3^Detroit Lions, Detroit, MI, United States; ^4^Toronto Raptors, Toronto, ON, Canada

**Keywords:** baseball, AIQ, athletic intelligence, major league baseball, cognitive assessment, intellectual ability assessment, professional sports, athletes

## Abstract

The focus on quantifiable data in sport performance has led to incremental advantages in baseball and has played an important role in the development of new hitting, pitching, fielding, and coaching strategies. Recently, researchers and team representatives have considered the impact of additional factors in baseball, including cognitive functioning. In this study, predictive validity for the Athletic Intelligence Quotient (AIQ) was examined vis-à-vis performance outcomes in professional baseball. Specifically, AIQ scores were obtained from 149 Minor League Baseball (MiLB) players prior to the 2014 baseball season and their subsequent performance was assessed through traditional and newly emphasized baseball statistics. Using hierarchical multiple regression, it was demonstrated that the AIQ predicted statistically significant relationships with hitting and pitching statistics, after controlling for other variables. Given the recent impact of analytics in professional sports, the potential importance of the AIQ in the selection and coaching process was discussed.

## Introduction

In Major League Baseball (MLB), the evaluation of athletic talent is a high stakes enterprise. According to a recent Forbes article (Ozanian, 2015), the average MLB team is now worth $1.2 billion. Considering the enormity of the baseball business, it is understandable that major efforts would be made to gain any advantage in player selection and development. However, even with significant time and money being spent, a solution to the puzzle of sport success has remained elusive.

In its history, sport performance research has included measurement of physical characteristics/abilities (e.g., anthropometric measures; Hoffman et al., 2009), personality constructs (e.g., coachability and mental toughness; Friend and Leunes, 1990), newly developed performance statistics (i.e., sabermetrics; Beneventano et al., 2012) and a range of cognitive factors. Though the relationship between cognitive functioning and sport expertise has been investigated widely, the specific factors studied and the manner in which they have been measured have varied considerably (Voss et al., 2010). As such, it has been difficult for organizations, coaches, practitioners, players, and others to fully understand the role of cognitive functioning in the game of baseball.

Nevertheless, several studies have recently made a strong case for the role of cognitive functioning in athletic performance (Fadde and Zaichkowsky, 2018; Brenton et al., 2019). For instance, in 2013 Faubert found that expert athletes were significantly better than amateur athletes and non-athletes in processing a non-sport-specific, complex dynamic visual task. In light of the growing research in this area, some have argued that the cognitive domain may, in fact, be the determining factor separating elite athletes (i.e., “playmakers”) from non-elite athletes (Zaichkowsky and Peterson, 2018).

In spite of the aforementioned research, cognitive assessments continue to be under-utilized by professional teams in the measurement of athletic talent. Further, the instruments that have been employed to date are limited in the information they provide. Perhaps the most well-known cognitive measure used in sports is the Wonderlic Personnel Test (WPT-R), a measure of general mental ability (GMA; Wonderlic Inc, 2002). However, the results of several research studies have indicated that scores from the Wonderlic test do not significantly predict performance outcomes in sports (Mirabile, 2005; Kuzmits and Adams, 2008). When taken together with studies on expert performance in other fields, such findings have led eminent researchers to conclude that measures of GMA have not yet demonstrated predictive power in sports (Ericsson, 2014). In fact, we agree with this point, but we believe that the failure of previous research to establish connections between measures of GMA and athletic performance has to do with which cognitive abilities have been measured (or not measured) in such research (e.g., Wonderlic).

In the field of cognitive assessment, there are several competing theories of intellectual abilities. However, few of the existing theories have obtained consistent empirical evidence for their foundational principles (Flanagan et al., 2013). According to multiple theorists and researchers, the theory of intelligence with the most supportive evidence is the Cattell-Horn-Carroll (CHC) theory of cognitive abilities (Alfonso et al., 2005; Flanagan et al., 2006). CHC theory has been widely investigated and applied in many fields. Its evidence base includes neurocognitive, developmental, and factor analytic research (Schneider and McGrew, 2018). Additionally, based on its strong empirical support, CHC theory has served as a foundation for significant revisions made to the most widely used intelligence and academic achievement tests (e.g., Wechsler Intelligence Scales for Children, 5th Edition; Alfonso et al., 2005; Flanagan et al., 2013).

Previous research has established correlations between specific CHC abilities and occupational success in a wide range of occupations (Dawis, 1994; Gardner, 1994; Lohman, 1994; Ackerman and Heggestad, 1997). Until recently, however, CHC theory had not been applied to the measurement of mental abilities and processes considered essential in elite athletes. One of the benefits of applying this theory to the domain of sports is that it provides a standard language that coaches, practitioners, and athletes can use to discuss the cognitive strengths and weaknesses of athletes. In addition, CHC theory provides a framework for conclusions drawn about athletes’ specific cognitive strengths and weaknesses, and these conclusions can be drawn with confidence.

It has been proposed that the CHC Theory of Intelligence may include as many as 18 broad cognitive abilities, which are each composed of several narrow abilities (Schneider and McGrew, 2018). Based on CHC Theory, not all intellectual abilities are expected to correlate with athletic performance; however, there are several that would be directly related. With this in mind, Bowman and Goldman created the Athletic Intelligence Quotient (AIQ). Four broad CHC abilities were chosen for inclusion in the AIQ: visual spatial processing, long-term storage and retrieval, reaction time, and processing speed (Bowman and Goldman, 2014).

Importantly, the AIQ does not include measures of other cognitive abilities that are more academic in nature (e.g., verbal knowledge, quantitative reasoning, etc.). This represents a significant difference in test composition when compared to existing measures of GMA (e.g., Wonderlic) and aligns the AIQ more with the more dynamic cognitive measures that have been shown to differentiate novice and elite athletes (Faubert, 2013).

Briefly, the AIQ was designed to assess “Athletic Intelligence,” in a manner consistent with the foundational principles of CHC Theory. According to Bowman et al. (2020), Athletic Intelligence includes the cognitive abilities that enable athletes to optimally visualize their surroundings in real time, learn and recall game information fluently, react quickly and accurately to stimuli, and sustain rapid decision making for extended periods. Thus, Athletic Intelligence is a highly specialized subset of previously identified and validated broad CHC abilities [i.e., visual spatial processing (Gv), Learning Efficiency (Gl), Reaction Time (Gt), and Processing Speed (Gs)]. Data from pilot research, reported in Bowman et al. (2020), was subjected to a confirmatory factor analysis which supported the AIQ being broken out into these four factors.

Initial validity evidence for the AIQ was established through a study in which athletes’ scores were compared to obtained scores on the Wonderlic Personnel Test and the ImPACT test (Lovell et al., 2000). The participants in this study included 93 Division 1 NCAA men’s lacrosse, men’s soccer, and women’s soccer players attending a northeast university (Crimarco et al., in preparation). Significant correlations were found between the visual spatial processing and long-term memory factors of the AIQ and the Wonderlic test, suggesting some overlap between these measures. Importantly, however, neither the Reaction Time nor the Processing Speed factors of the AIQ correlated significantly with the Wonderlic test, thereby demonstrating discriminant validity. Significant correlations were also found in expected directions between composites of the ImPACT and factors on the AIQ. For instance, the AIQ Reaction Time and Processing Speed factors correlated significantly with the ImPACT reaction time composite. These findings demonstrate convergent validity.

More recently, research has been undertaken to examine the relationships among AIQ factors and performance outcomes in the National Football League (NFL) (Bowman et al., 2020). Specifically, 146 NFL prospects were administered the AIQ at the 2015 and 2016 NFL Scouting Combines, and their scores were analyzed in relation to subsequent performance in the NFL. The results of this study revealed that specific AIQ factors accounted for a statistically significant increase in the explanation of variance in game statistics (e.g., rushing yards per carry) as well as overall ratings of player success (i.e., weighted career approximate value) beyond other important factors (i.e., draft order).

Finally, another study has recently demonstrated that players in the National Basketball Association (NBA) have significantly higher scores on three of the four factors of the AIQ, when compared to players in the G League or International Leagues (Hogan et al., in preparation).

There are other factors that impact performance in both athletics and measures of cognitive functioning. Within our targeted sample, two potential variables are age and country of origin, especially given the language barrier in the administration of such tests. To that end, although the AIQ was initially developed in English, it has since been translated into Spanish. The availability of Spanish-language cognitive measure is of critical importance when assessing the relationships between cognitive factors and athletic performance in baseball, as 26.5% of MLB players were Latino, as of opening day 2018 (Gentile and Buzzelli, 2021). Age effects have also been well documented in terms of cognitive development and decline (Salthouse, 2019). While not central to our main hypotheses, these variables were assessed to statistically control for potential confounds.

### Hypotheses

In light of the existing research on cognitive functioning and sport performance, we advanced the following hypotheses:

H1: The 4 factors of the AIQ (i.e., visual spatial processing, long-term storage and retrieval, reaction time and processing speed) would account for a statistically significant increase in the explanation of variance in traditional and new (sabermetric) hitting statistics (e.g., batting average, on-base plus slugging (OPS) percentage) beyond age, country of origin, and infield/outfield position.
H2: The 4 factors of the AIQ would account for a statistically significant increase in the explanation of variance in traditional and new (sabermetric) pitching statistics [e.g., earned run average (ERA), Fielding Independent Pitching (FIP)] beyond age and country of origin.
H3: Given the novelty of this data set, we also chose to explore the possibility of interactions among AIQ factors for both the pitching and hitting data, which could add to further variance explained by our models.

## Materials and Methods

### Experimental Approach to the Problem

The independent variables selected for inclusion in this study included AIQ factor scores, age, position, and country of origin. Age and country of origin were included to account for any differences resulting from diverse experiential/linguistic backgrounds (e.g., Latin American baseball academies) or cognitive development/decline. The specific dependent variables were chosen because they reflect performance outcomes in baseball. By controlling for the effects of age, position, and country of origin it was possible to identify the unique contributions of the AIQ in the prediction of professional outcomes in baseball.

### Subjects

A total of 149 Minor League Baseball (MiLB) players from a single major league organization were administered the AIQ prior to the start of the 2014 season. Performance statistics were then obtained at the conclusion of the 2014 season. Of the 149 athletes, 73 were position players and 76 were pitchers. Participants ranged in age from 19 to 37 (*M* = 24.6, SD = 3.14). The position players included 57 from the United States and 16 from Latin America. The pitchers included 66 players from the United States and 10 from Latin America. Access to this sample was a unique opportunity and we collected as many cases as the opportunity would allow. Power analyses using G^∗^Power (Faul et al., 2007) suggested that we had power >0.90 for detecting medium effect sizes given our intended regression analyses.

### Instruments

#### Athletic Intelligence Quotient

The athletic intelligence test is a measure of cognitive ability composed of 10 subtests (see Table 1 for subtest descriptions and reliability coefficients). At the time of this study, it was individually administered by a software program on the Samsung Galaxy Tab, with the ice cream sandwich version of the android operating system. Subtests are presented in a fixed, successive order, with audio/visual instructions provided before the start of each task. The administration time for the AIQ generally ranges from 35–38 min. Resulting scores on the AIQ include a Full Scale AIQ Score (FSAIQ), four factor scores (i.e., visual spatial processing, reaction time, processing speed, and learning efficiency), and 10 subtest scores. In order to minimize the likelihood of Type I error, in this study, only the four factor scores were analyzed with respect to performance outcomes in baseball. See the AIQ Professional Manual for information about each subtest and factor (Bowman and Goldman, 2014). More detailed information about the development of the AIQ and evidence of its validity with respect to athletic performance data is available in Bowman et al. (2020).

**TABLE 1:** AIQ subtest descriptions.

**Subtest**	**CHC narrow/broad ability**	**Reliability**	**Description**
**Shape rotations**	Visualization/visual spatial processing	0.77 Test-re-test	Measures the ability to mentally rotate shapes in one’s mind and see how they would look under different circumstances. In particular, examinees are presented with a given target shape and they must decide whether the shapes below it are the same (only rotated) or are different and would need to flipped over to look the same.
**Paired associative learning**	Associative memory/learning efficiency	0.91 Internal consistency	Assesses the ability to form a mental link between random stimuli. In particular, the examinees are presented with 16 pictures that have been paired with random two-digit numbers. They are shown each pair for 2 s before having to provide the missing two-digit numbers when presented with the pictures alone. This procedure is then repeated for a two additional trials.
**Object scanning**	Perceptual speed/processing speed	0.81 Test-re-test	A cancelation task measuring the ability to quickly scan a visual field to locate 3 target shapes among both targets and distractors.
**Route finding**	Spatial scanning/visual spatial processing	0.57 Test-re-test	Assesses the ability to find the shortest route between two locations as quickly as possible, while having to avoid obstacles.
**Simple reaction time**	Simple reaction time/reaction time	0.79 internal consistency	Examinees are instructed to press a button as fast as possible after a stimulus (i.e., square) appears on the screen. When the response key is pressed, the square disappears from the screen. If the response key is not pressed within 1,000 ms of the presentation of the square, it will automatically disappear. The time between presentations of the square (viz., interstimulus interval) varies between 500 and 2000 ms. The subtest scores are based on both speed and accuracy, with omissions and commissions resulting in lower scores.
**Memory for shapes**	Visual memory/visual spatial processing	0.90 Internal consistency	Assesses visual memory by asking examinees to study an array of 16 shapes. Next, the examinees are presented with each of the original shapes, but they are out of order on the bottom of the screen. They must then drag the shapes to their correct locations.
**Number matching**	Perceptual speed/processing speed	0.81 Test-re-test	On this task, two multi-digit numbers are presented side-by-side on the screen. The examinee must indicate whether the two numbers are the same or not. The examinee has 2 min to make as many comparisons as possible.
**Choice reaction time**	Choice reaction time/reaction time	0.77 Internal consistency	Assesses reaction time and detectability by presenting two target stimuli and three distracter stimuli in random order. The examinee must press the response key as quickly as possible when presented with one of the two target stimuli, but must refrain from pressing the key when any of the three distracters are presented. If the response key is pressed, the image is removed. If the key is not pressed, the image disappears after 1,000 ms. Again, the resulting subtest scores are based on both speed and accuracy, with omissions and commissions resulting in lower scores.
**Design matching**	Spatial relations/visual spatial processing	0.84 Test-re-test	Examinees are shown a design at the top of the screen and they must replicate the design by touching empty boxes until each one matches the stimulus.
**Paired associative learning delayed**	Associative memory/learning efficiency	0.83 Internal consistency	This subtest is administered approximately 30 min after the first paired associative learning task is given. It assesses the examinee’s ability to recall the information learned from the three previous trials.

#### Baseball Performance Measures

Hitting and pitching statistics from all MiLB players who took the AIQ were collected by one MLB team throughout the 2014 baseball season. Although these statistics are publicly available, they were compiled by an MLB team, who was tracking the performance of their minor league players. This MLB team then made the performance statistics available to the authors.

Season statistics included: batting average (AVG), slugging percentage (SLG), OPS, Isolated Power (ISO), walks plus hits per inning pitched (WHIP), ERA, and FIP. Batting average is the number of hits obtained per at-bat. SLG represents the total number of bases a player records per at-bat. It differs from batting average in that all hits are not valued equally. OPS is the sum of a player’s on-base average and their SLG. ISO is a player’s SLG minus their batting average. WHIP is self-evident. ERA is the number of earned runs allowed per 9 innings pitched. Finally, FIP is similar to ERA, but it focuses solely on the events a pitcher has the most control over – strikeouts, unintentional walks, hit-by-pitches and home runs. It entirely removes results on balls hit into the field of play. The means and standard deviations for these performance statistics are included in Table 2.

**TABLE 2:** Means and standard deviations of baseball statistics.

**Hitting statistics (*N* = 73)**	**M**		**SD**	
AVG	0.25		0.03	
Batting average				
SLG		0.37		0.06
Slugging percentage				
OSP	0.70		0.08	
On base plus slugging				
ISO	0.12		0.04	
Isolated power				
Composite hitting measure		0.0		0.90
**Pitching statistics (*N* = 73)**				
ERA	3.73		1.11	
Earned run average				
WHIP		1.34		0.26
Walks plus hits per inning				
pitched				
Fielding independent pitching (FIP)		3.22		0.74

### Procedures

The assessment protocol was briefly described before participants were asked to provide informed consent, which included their express right to discontinue responding to assessment questions at any time. When the athletes arrived at the evaluation room, they were individually led to the testing station by a trained administrator who briefly explained the testing procedures. Next, an examiner initiated the computer program for the participants and presented them with headphones for audio instructions.

## Results

### Hitting Data

To obtain a full picture of players’ hitting abilities, we used the following measures: Batting AVG, SLG, OPS, and ISO. As shown in Table 3, these measures were highly correlated; subsequently, we ran a reliability analysis, which yielded a Cronbach’s alpha level of 0.88, suggesting that these separate scores were reliably assessing the same underlying construct. For the sake of parsimony, we created a single composite measure standardizing all four hitting measures, then averaging across the standardized values for each player. This composite measure was then entered into a hierarchical multiple regression as the dependent variable, using the following model: block 1: age of athlete, whether the athlete was from the US or not; block 2: whether the athlete played in the infield or outfield; block 3: the four factors of the AIQ – visual spatial processing, processing speed, reaction time, and learning efficiency; block 4: the interactions between the four factors of the AIQ. Our exploratory analysis for the interaction terms of the AIQ yielded no significant results, so we dropped that block and re-ran the regression with just blocks one through three. Overall, the full model was significant, *R*^2^ = 0.27, *F*(7,62) = 3.27, *p* = 0.005, but since neither reaction time nor learning efficiency were significant, these two variables were trimmed from the final model.

**TABLE 3:** Descriptive statistics and zero-order correlations for hitting variables and AIQ measures (*N* = 73).

	***M***	**SD**	**AVG**	**SLG**	**OPS**	**ISO**	**Visual spatial processing**	**Reaction time**	**Processing speed**	**Learning efficiency**
Hitting	0	0.9	0.83**	0.99**	0.96**	0.86**	0.24*	0.16	0.08	−0.01
Composite										
Measure										
AVG	0.25	0.03		0.78**	0.82**	0.41**	0.22^†^	0.20^†^	0.21^†^	0.03
SLG	0.37	0.06			0.91**	0.89**	0.24*	0.14	0.06	−0.02
OPS	0.70	0.08				0.72**	0.24*	0.17	0.10	0.00
ISO	0.12	0.04					0.19	0.06	−0.07	−0.05

^†^*p* < 0.10, *p < 0.05, **p < 0.01.

Table 3 provides the descriptive statistics of the composite hitting variable and each of the predictor variables, along with the zero-order correlations. Of note is that the visual spatial processing factor was significantly correlated with the hitting composite measure, as well as SLG and OPS, and was marginally correlated with AVG. Using a standardized composite measure to assess hitting, essentially creating a latent variable, increases the power of the analyses and avoids repeating the same essential finding across four strongly overlapping measures. Regression analyses on the component measures shows mostly the same pattern except for batting average. The pattern of the component measures are best captured by the zero order correlations which speak to the impact of the AIQ measures on the hitting metrics. The other factors of the AIQ did not demonstrate significant correlations with these hitting statistics.

Table 4 shows the results of the regression analysis, broken out by hierarchical order of entry. Age of athlete was marginally positively correlated with better hitting, being from the United States was associated with better hitting and outfielders had better hitting than infielders. Among the significant AIQ measures, better visual spatial processing was associated with better hitting, but slower processing speed yielded better hitting. These last two variables demonstrate the utility of the AIQ with its four factors and basis in CHC theory, explaining an additional 7.6% of the variance in hitting, above and beyond the descriptive variables of age, country of origin, and field position.

**TABLE 4:** Hierarchical regression of hitting composite measure as a function of age, country of origin, field position dichotomy, and AIQ factors.

**Step and predictor variable**	*R*^2^	Δ*R*^2^	*sr*^2^	β
Step 1	0.12*	0.12*		
Age			0.19	0.19*
Country of origin			−0.25	−0.25*
Step 2	0.17**	0.05*		
Field position: infield or outfield			−0.23	−0.22*
Step 3	0.24**	0.08*		
Visual spatial processing			0.31	0.23*
Processing speed			−0.34	−0.24*

*p < 0.05. **p < 0.01. ***p < 0.001.

### Pitching Data

To evaluate pitching data, we examined the variables of ERA, WHIP, and FIP, starting with an evaluation of their intercorrelations. Although not as large in magnitude as the hitting data, these measures were moderately to strongly correlated. Based on this finding, we decided to evaluate these measures separately in a series of parallel hierarchical multiple regressions with the following model: block 1: age of athlete, whether the athlete was from the US or not; block 2: the four subscales of the AIQ – visual spatial processing, processing speed, reaction time, and learning efficiency; block 3: the interactions between the 4 factors of the AIQ. Neither of the analyses focused on the WHIP and the FIP yielded significant results; however, the analysis of ERA demonstrated a significant overall model, *R*^2^ = 0.35, *F*(12,58) = 2.62, *p* = 0.007. Due to non-significant findings, processing speed, learning efficiency, and all non-significant interaction terms of the AIQ measures were trimmed from the model.

Table 5 provides the descriptive statistics of the three pitching measures and each of the predictor variables, along with the zero-order correlations. Reaction Time was significantly negatively correlated with ERA, and marginally correlated with WHIP. Additionally, long-term efficiency was significantly positively correlated with FIP.

**TABLE 5:** Descriptive statistics and zero-order correlations for pitching variables and AIQ measures (*N* = 76).

	***M***	**SD**	**WHIP**	**FIP**	**Visual spatial processing**	**Reaction time**	**Processing speed**	**Learning efficiency**
ERA	3.73	1.13	0.77**	0.57**	−0.11	−0.33**	−0.03	0.09
WHIP	1.34	0.26		0.72**	−0.03	−0.23^†^	−0.01	0.14
FIP	3.22	0.74			0.16	−0.06	0.12	0.26*

^†^*p* < 0.10, *p < 0.05, **p < 0.01

**FIGURE 1:**
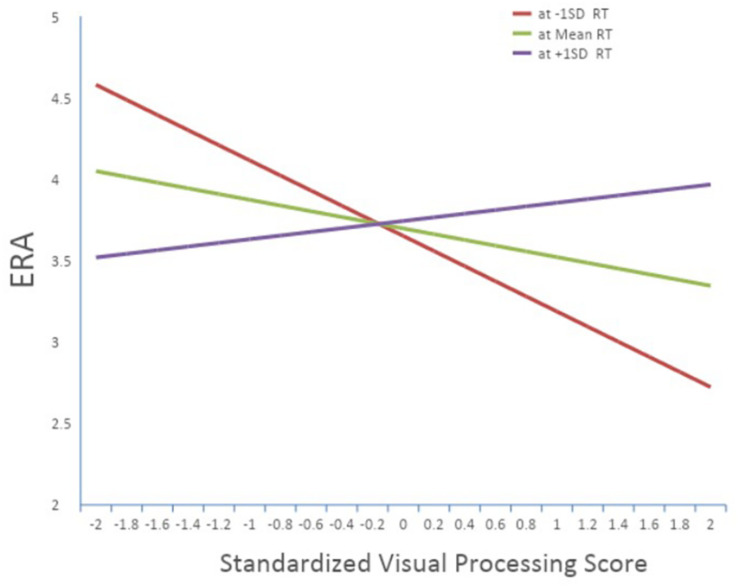
Interaction of visual spatial processing with reaction time on ERA.

Table 6 shows the results of the regression analysis of ERA, broken out by hierarchical order of entry. The pattern of results did not emerge until the final step of the model, but in earlier steps age and reaction time were marginally related to ERA and RT was negatively related to ERA. However, these main effects were qualified by a significant interaction between visual spatial processing and RT. As shown in Figure 1, when Reaction Time is slow (1SD below the mean RT), higher visual spatial processing is related to better (lower) ERA, whereas when RT is faster (1SD above the mean RT), lower visual spatial processing is related to better ERA. Once again, as shown in Table 6, a combination of the AIQ measures explained a statistically significant 20% of the variance in pitching (ERA), above and beyond the descriptive variables of age and country of origin.

**TABLE 6:** Hierarchical regression of ERA as a function of age, country of origin, and AIQ measures.

**Step and predictor variable**	***R*^2^**	**Δ*R*^2^**	***sr*^2^**	**β**
Step 1	0.07	0.07		
Age			0.07	0.07
Country of origin			0.27*	0.28
Step 2	0.15**	0.08*		
Visual spatial processing			−0.09	−0.11
Reaction time			−0.24*	−0.25
Step 3	0.28***	0.13***		
Interaction between visual spatial			0.36***	0.42
Processing and reaction time				

*p < 0.05. **p < 0.01. ***p < 0.001.

## Discussion

Previous research has demonstrated some success in utilizing sabermetrics, measures of physical abilities, personality measures, and cognitive factors to predict players’ performance outcomes in baseball. The current research adds to the growing body of research on cognitive factors in baseball by investigating a state-of-the-art assessment based on the CHC Theory of Intelligence. To that end, specific aspects of cognitive functioning were predictive of hitting, with models based upon age, country of origin and AIQ factors explaining 27% of the variance in performance.

Notably, the measures of visual spatial processing and processing speed of the AIQ were significant predictors of hitting. This relationship makes intuitive sense, as hitters must process factors such as the trajectory, spin rate, and location of incoming pitches. They also need to be cognizant of their own body mechanics and maintain proper orientation and spacing as they swing. A player’s visual spatial processing and processing speed would likely impact each of these skills. Additionally, visual spatial processing, processing speed, and reaction time each demonstrated marginally significant correlations with batting average, in particular. Reaction time, too, would seem to be an important factor for hitters, especially since pitches traveling at 100 mph take just 400 ms to travel from the pitcher to the hitter. This does not leave much time for the player to engage the skills above, make the snap judgment to swing or not, and then swing the bat.

With respect to pitching, cognitive factors were also predictive, with models including age, country of origin and AIQ accounting for 32% of the variance in ERA. In particular, reaction time was significantly correlated with pitchers’ ERA and marginally correlated with WHIP. One may not necessarily think of reaction time as being quite as important in pitching as it is in hitting, because the delivery to the plate is largely a self-timed action. However, it is possible that reaction time plays a role in other important aspects of pitching, such as fielding one’s position, covering bases, and holding runners on base. Each of these parts of the game would necessitate immediate processing of information. Additionally, the reaction time factor of the AIQ may tap elements of a player’s broader executive functioning, which may also contribute to their performance.

Overall, the results from this study are consistent with previous findings in that elite athletes show a superior advantage in decision-making and problem-solving (Voss et al., 2010; Jacobson and Matthaeus, 2014). Presumably there is some natural advantage for these athletes, which then get honed over the course of experience. Further, some of the evidence suggests that a distinct skill set may be developed, relating specifically to baseball skills (Nakamoto and Mori, 2008). The current results add to the existing literature by placing the cognitive skills within a broader context of cognitive assessment, namely CHC Theory. Further, the current results also account for some significant demographics and include interactions that have not been previously assessed.

Specifically, the significant interaction between visual spatial processing and reaction time for pitchers indicate that there may be different cognitive mechanisms at play that contribute to a pitcher’s success on the mound. In fact, the pattern we found suggests two avenues to a lower ERA: one in which pitchers rely more on their strong visual spatial processing if their reaction time is slow, and conversely, one in which pitchers rely more on their immediate processing of information (i.e., reaction time) if their visual spatial processing is poor. It is possible that this interaction effect reveals different processes for pitchers who rely more on effective pitch location as opposed to the velocity and/or movement of their pitches.

The current research also provides insight into the interrelationships between the various measures of successful performance in MLB players. In particular, as noted in Table 2, there is considerable overlap between the various sabermetrics, particularly those relating to hitting. This overlap was so great that we elected to combine these stats into a single composite measure. Typically this statistical technique yields the best overall assessment since any error loading on one measure is canceled out by error loading on any other measures. However, it is clear that these different measures were largely interchangeable in this study.

As with all research, there were limitations. The current sample relied solely on demographics such as age, position, and country of origin and the cognitive measures assessed in the AIQ. The variance predicted by these measures appears to be higher than that of previous efforts to predict performance based upon cognitive assessment, but without a direct comparison within the sample, it is impossible to assert definitively that one set of cognitive measures is significantly better than another. Further, the inclusion of other factors, such as physical prowess, deliberate practice, and personality may also capture some of the variance explained by the AIQ measures in the sample. Conversely, by holding some of these other factors constant, it is possible that more significant findings could be found with the AIQ.

Future research should look to replicate and extend these findings. With a good deal of the variance in performance still unexplained, there is considerable room for developing a model with even greater predictability. Such improvement could come from increased sample sizes, allowing a greater focus on the different positions. A wider range of measures offers the possibility of either consolidation of predictive power or greater predictive power, depending on how much overlap exists between the various domains of performance, personality, and cognitive factors. It also would be possible to compare which of the 10 subtests of the AIQ yield the most utility in making predictions, which could help tailor assessments more specifically to the game of baseball, as the AIQ was designed for use across multiple sports.

Ultimately, even though a solution to the puzzle of sport success is likely to remain elusive indefinitely, the current findings suggest that the measurement of specific cognitive abilities contributes to a better understanding of performance in professional baseball. As teams work to strategically draft and develop players in this high-stakes game, it would appear that improved understanding of players’ athletic intelligence would be advantageous.

## Practical Applications

Based on the findings from the present study, there appears to be a growing evidence base for the validity of the AIQ (Bowman et al., 2020; Crimarco et al., in preparation; Hogan et al., in preparation). As the results of this investigation suggest, strong cognitive abilities alone cannot necessarily compensate for differences in physical skills, work ethic/deliberate practice, or personality functioning in terms of performance outcomes. However, knowledge of athletes’ cognitive strengths and weaknesses still serves several important purposes. Perhaps most importantly for practitioners, it may help them find a goodness of fit in coaching/player development strategies to optimize outcomes for the athletes.

As an example, if practitioners are able to identify that aspects of a hitter’s visual spatial processing are weak, it may lead to exploration of strategies designed to enhance their understanding and recognition of the trajectory, spin, or location of a pitch. It could also lead to new ways of helping the hitter perceive his body in space, to improve consistency in his physical mechanics at the plate. For those in the sport psychology and strength and conditioning fields, it is often critical to identify how athletes think, learn, and process information. Thus, there is a clear need for a valid and reliable cognitive assessment, such as the AIQ, that can be used to help practitioners better understand and assist their athletes.

## Data Availability Statement

The raw data supporting the conclusions of this article will be made available by the authors, without undue reservation.

## Ethics Statement

Ethical review and approval was not required for the study on human participants in accordance with the local legislation and institutional requirements. The patients/participants provided their written informed consent to participate in this study.

## Author Contributions

JB assisted with literature reviews, data collection, statistical analyses, and manuscript writing. RB assisted with statistical analyses, manuscript writing, and editing. SG assisted with literature reviews, data collection, and manuscript writing and editing. AA assisted with data collection and manuscript writing and editing. All authors contributed to the article and approved the submitted version.

## Conflict of Interest

In addition to his role as a school psychologist for the Great Neck Public Schools, JB is a co-creator of the AIQ and partner at Athletic Intelligence Measures (AIM), LLC. SG is a sport psychologist for the Detroit Lions, but he is also a co-creator of the AIQ and partner at AIM, LLC. AA is a sport psychologist for the Toronto Raptors, but he is also a consultant for AIM, LLC. The remaining author declares that the research was conducted in the absence of any financial or commercial relationship that could be construed as a potential conflict of interest.
